# Mental health professionals view about the impact of male gender for the treatment of men with depression - a qualitative study

**DOI:** 10.1186/s12888-020-02686-x

**Published:** 2020-06-03

**Authors:** Maja Stiawa, Annabel Müller-Stierlin, Tobias Staiger, Reinhold Kilian, Thomas Becker, Harald Gündel, Petra Beschoner, Achim Grinschgl, Karel Frasch, Max Schmauß, Maria Panzirsch, Lea Mayer, Elisa Sittenberger, Silvia Krumm

**Affiliations:** 1 Department for Psychiatry and Psychotherapy II, Ulm University at BKH Guenzburg, Ludwig-Heilmeyer-Str. 2, 89312 Guenzburg, Germany; 2 Department of Psychosomatic Medicine and Psychotherapy, Ulm University, Ulm, Germany; 3 Department for Psychosomatic Medicine and Psychotherapy, Günztalklinik Allgäu, Obergünzburg, Germany; 4 Department of Psychiatry, Psychotherapy and Psychosomatic Medicine, BKH Donauwörth, Donauwörth, Germany; 5 Department of Psychiatry, Psychotherapy and Psychosomatic Medicine, Augsburg University, Augsburg, Germany; 6 Department of Psychiatry and Psychotherapy III, Ulm University, Ulm, Germany

**Keywords:** Depression, Men’s depression, Qualitative research, Subjective view, Masculinity

## Abstract

**Background:**

The underestimation of depression among men may result from atypical depression symptoms and male help-seeking behaviour. However, higher suicide rates among men than among women indicate a need for gender-specific services for men with depression. In order to develop gender-specific services, it is essential to examine professionals’ attitudes towards men’s depressive symptoms and treatment needs as well as barriers to and facilitators of treatment. This study examined gender-specific treatment needs in male patients and treatment approaches to male patients from a professional perspective.

**Methods:**

Semi-structured face-to-face interviews were conducted with 33 mental health professionals (MHPs) from five German psychiatric institutions. The study assessed the characteristics and attributes of male patients with depression risk factors for the development of depression among men, their condition at the beginning of treatment, male patients’ depressive symptoms, the needs and expectations of male patients, the importance of social networks in a mental health context, and MHPs’ treatment aims and treatment methods. Transcripts were analysed using qualitative content analysis.

**Results:**

The professionals’ reference group of male patients were men who were characterised in accordance with traditional masculinity. Attributes reported as in line with this type of men were late initiations of inpatient treatment after crisis, suicidal ideation or attempted suicide, and high expectations towards treatment duration, success rate in recovery and therapeutic sessions. In contrast, male patients who deviate from these patterns were partially described with reference to female stereotypes. Professionals referred to psychosocial models in their explanations of the causes of depression and provided sociological explanations for the development of masculine ideals among men. The consequences of these for treatment were discussed against the background of normative expectations regarding the male gender. From the professionals’ point of view, psychoeducation and the acceptance of depression (as a widespread mental illness) were the most important goals in mental health treatment.

**Conclusions:**

In order to improve mental health among men, gender-specific services should be offered. Awareness of the role of gender and its implications on mental health treatment should be an integral part of MHPs’ education and their daily implementation of mental health treatment practices.

## Background

Women are twice as likely to be diagnosed with depression during their lifetime as men [1–3]. This gender disparity is believed to occur for both biological and psychosocial reasons: for example, hormonal changes are assumed to increase women’s vulnerability to depression at particular points in their lives, such as during adolescence [4] or after giving birth [5, 6]. In addition, women are perceived as being more likely to be exposed to social hardship such as poverty or single parenting, resulting in adverse effects on their mental health [7–11]. At the same time, suicide rates among men worldwide are higher than among women [12]. The discordant relationship between men’s low rates of diagnosed depression and high male suicide rates calls for an enhanced detection and treatment of depression among men [13, 14]. According to the concept of male depression, men tend to experience externalized symptoms such as aggression and substance abuse more often, whereas women tend to more regularly experience internalized symptoms [15–18]. The presence of externalized symptoms was found to decrease men’s capacity to seek help and lead to less favourable depression care compared with men who show internalized symptoms [19]. In addition, studies have found evidence which suggests that diagnostic instruments that focus on internalized symptoms lead to an underestimation of depression levels among men. Studies have shown that gender differences in terms of the prevalence of depression converge with the inclusion of externalizing symptoms in diagnosis practices [20, 21]. Furthermore, the lower prevalence of depression among men has also been discussed as resulting from men’s reluctance to seek help for depressive symptoms, behaviour which is in line with male gender norms [22]. A number of qualitative studies on men’s views on mental health provide evidence that shows they assess depression as being incompatible with male gender role expectations [23, 24], associate it with female attributes like weakness [25, 26] or do not take it seriously as an illness [25].

Due to gender-based differences in rates of depression, high suicide rates among men, and male-specific help-seeking behaviour, there is a strong need for gender-specific services tailored to men with depression. A recently-published report on men’s health by the World Health Organization emphasized the impact of gender norms on men’s health and the need for new strategies to address unmet mental health needs and improve mental health among men [27].

MHPs are in a central position to consider the gender-specific needs of service users and deliver services that reflect these. Due to the paradigm of social constructivism, gender is rather a result of social interaction than biologically determined. Due to the assumption of “doing gender”, people experience, attribute und act in accordance with their (unconscious) perceptions of gender [28]. Against this background, it is essential to examine attitudes and views of MHPs toward gender when exploring processes of mental health treatment in men with depression.

One qualitative study explored treatment experiences of men with depression and deduce recommendations for depression treatment [29]. While they found that action oriented, solution- focused and transparent treatment approaches are likely to maintain treatment engagement in men in general, the authors emphasize the importance to employ treatment approaches that consider different expressions of masculinities and build on the strengths of each form of masculinity to raise attractivity to male patients [29]. To date, there is very limited knowledge on how mental health (MH) services are sensitive towards and deal with the treatment needs of men with depression. In a qualitative study, Apesoa-Varano and colleagues described various treatment approaches used by MHPs in order to meet treatment needs in older men with depression [30]. There is also some evidence that suggests the existence of gender stereotypes among MHPs that have consequences for MH treatment. Oute and colleagues (2018) have pointed out that MHPs’ attribution of gender characteristics to patients determines patients’ access to mental health services and therefore has an impact on mental health outcomes [31]. Harris and colleagues (2001) explored the role of gendered assumptions and client sex in couple’s therapy and came to the result that therapists perceive patients’ behaviour differently depending on patients gender [32]. Other study results suggest that the gender of the patient has an impact on treatment success. Owen and colleagues (2009) explored the relationship of patients’ gender and treatment outcomes and showed varying gender competencies in therapists depending on patients’ sex. While some therapists reached the same treatment results with patients of each sex, others raised better results with male or female patients respectively [33]. Other research found evidence to suggest that MHPs concept of illness has consequences for therapeutic approaches and treatment strategies used in MH services. Ahn and colleagues (2009) explored mental health clinicians’ beliefs about the causes of mental health problems as psychologically, environmentally or biologically based [34]. They found that mental health clinicians rather consider one of these reasons to be a main cause for mental health problems depending on the illness, while contributory causes where considered less important. Clinicians further proposed various treatment approaches depending on the illness, estimating medication more reasonable for biologically based disorders and psychotherapy for psychosocially based disorders [34].

In order to identify barriers to and facilitators of the provision of gender-specific mental health services, it is essential to get a broader understanding of MHPs’ views on men’s needs and the implications of these for the treatment of depression in men. This study aims to shed light on gender-specific treatment of depression in Germany from a professional perspective by exploring MHPs’ views on (1) depression in men, (2) male patients’ needs, and (3) therapeutic approaches and treatment strategies regarding men with depression.

## Methods

This qualitative study was part of a mixed-method study titled “Masculinity Constructions and Mental Health Behaviour among Men with Depression” funded by the German Research Foundation (Project nr. 288,917,560). This paper addresses MHPs’ perspectives on the treatment of men with depression. The study was approved by the ethics committee of Ulm University (Nr. 202/15).

### Recruitment

MHPs were recruited in five psychiatric hospitals in a rural area of southern Germany.

In Germany, psychiatric hospitals represent an essential type of care. In general, inpatient treatment consists of psychotherapeutic treatment and medical treatment, supplemented by various types of alternative therapies, depending on the orientation of the respective hospital. Treatment in psychiatric hospitals is available to all patients in Germany if needed and covered by the statutory health insurance, which insures a large part of the population (about 90%). The total number of staff in all hospitals ensures a large sample for recruitment of MHPs and a wide range of experiences and expertise of staff with treatment of men with depression. Staff members of all professional groups (physicians, psychologists, nurses and social workers) were informed about the study and were invited to participate. In these hospitals, the study was presented at clinic conferences, in order to reach psychiatrists and psychologists, as well as during team meetings for nurses and social workers. Staff members of all professional groups were informed and asked to participate via e-mail, in three stages. MHPs who were interested in taking part in the study contacted the researchers to set the time for the interview. Participant recruitment was stopped at the point at which no new information could be obtained from the interviews.

### Data collection

Between May 2017 and February 2018, 33 interviews were conducted by two female researchers, both experienced in qualitative research. Participants received detailed information and provided written informed consent. A short questionnaire was used prior to the interview to collect information about participants’ profession, age and professional experience. A semi-structured interview guide was developed around the specific characteristics and attributes of male patients with depression, risk factors regarding the development of depression among men, their condition at the beginning of treatment, depressive symptoms among men, the needs and expectations of male patients, the importance of social networks in a mental health context, and MHPs’ treatment aims and treatment methods (Additional file 1).

To develop the interview guide, a carefully literature research was conducted as a first step by the first author (MSt). Afterwards, important aspects regarding the inpatient treatment of men with depression were identified by the first author and frequently discussed with the last author (SK). The interview guide was first pilot-tested with one physician.

The interview guide was followed to various degrees, for example with regard to question order, depending on the topics that were addressed by participants.

If participants did not distinguish between different types of men on their own, researchers explicitly asked for them to identify differences between individual male patients. If participants did not distinguish clearly between male and female patients, researchers asked for differences between men and women generally for the sake of providing a more significant distinction. Interviews were conducted at MHPs’ workplace. The duration of interviews varied between 19 and 44 min, with a mean of 32 min. All interviews were audio-recorded and transcribed verbatim in German. All transcripts were checked for accuracy by a student research assistant.

### Analysis

Content analysis was conducted in line with Kuckartz [35]. Firstly, all transcripts were carefully read and annotated. In order to get a good overview about the material, the first author wrote a case summary for every single case, structured around the different sections of the interview guide. Afterwards, a preliminary analysis was conducted by the first author and a system of categorization developed based on the results of five interviews. As the first step of these analysis, main categories were developed. In line with Kuckartz, main categories might derive from the research question or the interview guide respectively [35]. Thus, the interview guide served as initial template for deductive codes.

In the following step, text sections of the first five interviews were coded by main categories. Text sections were assigned to several main categories if there was more than one main category suitable. Thereafter, all material assigned to a main category was structured by developing sub-categories by inductive coding procedures. During these processes, 39 sub-categories (with further subdivisions also occurring) were developed.

After a preliminary system of categories was developed on the results of five interviews, all material was analysed in detail using MAXqda 11 software. During the analysis, the categories continuously evolved by modifying, summarising or deleting. In this process, main categories and sub-categories were further refined. Categories were deleted when they turned out to be irrelevant. E.g. the deductive category “sex of MHP” turned out as not relevant for participants. Contrary to this, the category “MHPs descriptions of male patients” emerged as an inductive category throughout the process of analysis. Descriptions of male patients appeared in different interview sections including participants’ statements on men’s illness concepts, circumstances of treatment start and treatment itself. While in the beginning of the process, the category was labelled as “types of men” including no sub-categories, this category was relabelled “differences between men” containing several sub-categories (the “typical” men, the “nontypical” men, dealing with the illness, external differences, and environment). The final code “MHPs description of male patients” is presented in Table 2.

In order to ascertain the relevance of each respective issue, the frequency of codes in total and the number of MHPs who referred to the respective issue was checked by the first author (MSt). Issues were coded by sentence. In cases when themes occurred several times in one sentence or if participants referred to a theme throughout a section without a break, themes were coded just once as well.

The analysis was documented in a research diary by the first author and presented and discussed in a weekly team meeting (SK, TS, MSt) to ensure consensual coding. In order to achieve a joint consensus, the manuscripts of five of the interviews were read and coded independently by SK, TS and MSt and talked over section by section to develop a preliminary coding system. If different opinions on the coding arised, interview sections were discussed until joint consensus was achieved. Additionally, the progression of the analysis and preliminary results were frequently presented and discussed in qualitative research workshops.

### Sampling characteristics

The 33 participating professionals differed with regard to age, years of professional practice in psychiatry, hospital affiliation, and profession. The participants consisted of seven physicians, nine psychologists, 11 nurses, one therapist and five social workers, and came from five different hospitals. Twenty-two of the participants (66%) were female. Participants’ ages ranged from between 25 to 61, with an average of 44. Participants’ years of practice in psychiatry varied from between half a year to 38 years, with 14 years the average for both male and female participants (Table 1).

**Table 1:** Participant Characteristics (*n* = 33)

Gender (n)	Male (11)	Female (22)
Age (M)	29–61 (45)	25–61 (44)
Years of practice (M)	0.5–38 (14)	0.5–37 (14)
Profession groups (n):		
Physician	5	2
Psychologist	2	7
Nurse	3	8
Social worker	0	5
Therapist	1	0

## Results

Five central themes were derived from the analysis: 1.) MHPs’ descriptions of male patients; 2.) MHPs’ theory of illness; 3.) Admission into the mental health care system; 4.) MHPs’ views of male patients’ expectations and 5.) MHPsʼ mental health treatment aims. These main themes contained 19 codes and 48 subcodes (Table 2). Participants are identified by their gender, profession and age.

**Table 2:** Coding tree

Theme	Code	Subcode	Quantity		Examples
			Person	Quote	
MHPsʼ description of male patients	The “typical” man	Behaviour	11	19	“reserved”
		Appearance	1	1	“bald head”, “beard”, “rather muscular”
		Attributes	18	25	“man of action”
		Cultural background	9	9	“If someone comes from Turkey or from Afghanistan, then mental illness is very taboo, you can’t have that there.”
		Formal education	2	2	“But [men who are, M.S.] somehow simply structured, and poorly educated; in the beginning, they tend to only scratch the surface.”
		Urban/rural area	5	6	“I would guess, until people who live in rural areas can admit to themselves that they are sick, I think this will take much longer. Because everyone knows everyone.”
		Age/generation	14	24	“So particularly the older generations, these men are doers. And they do. And they get through it.”
	The “non-typical” man	Behaviour	5	9	“talkative”, “unsure”, “afraid”,“passive”, “shy”
		Appearance	1	2	“long hair”, “weedy”
		Attributes	7	15	“softy”, “to be led by one’s head”, “wimp”
		Cultural background	1	1	“If someone comes from Sweden or Denmark, then it’s a similar context to ours.”
		Formal education	7	8	“[...] with an academic who’s depressed, he’s got a little more access to his emotional world.”
		Urban/rural area	1	1	“It is probably easier for people in the city to seek treatment.”
		Age/generation	8	11	“And the younger they are, the more open they are somehow.”
	No gender variation		14	26	“This is also very dependent on the individual. I think it depends on the make-up of their families.”
MHPsʼ theory of illness	Mental illness in media and society		14	25	“Football coaches also get burnout and depression; football players too. And I believe that society has come to accept that people do not have limitless resilience.”
	Normative superstructure	Change in society leads to overload	4	5	“That this may well cause problems, in modern or postmodern times, to find oneself in a role or in multiple roles, and because of much higher demands and choices, the risk of failure is much greater.”
		(successful) employment expected	14	19	“It is simply a reflection of societal attitudes that men are still expected to be employed and to want to be employed, so to speak.”
		Depression is related to weakness/failure	10	14	“I’m big, I’m strong, I take care of others. I can do anything, I’m a somebody. I’m not sick. I’m not allowed be sick. I am the strong one.”
	Reason / occasion of depression	Missing/poor coping strategies	17	30	“And they just escape, at least that’s my experience, often into drugs or alcohol.”
		Personality	10	18	“And these are also more likely men who normally present as strong personalities, and perhaps also with narcissistic elements.”
		Socialisation	17	36	“These are often defining childhood experiences; the family situation during childhood and youth, the parents as role models or supporting caregivers.”
		Family burden	12	13	“Or that they are overwhelmed by their family situations.”
		working conditions	30	74	“A frequent problem tends to be the workplace, with excessive demands in their job and the threat of job loss or being humiliated in the workplace.”
		Relationship problems	19	32	“In my experience with men, family problems in particular crop up again and again as well; following separation, and also during a divorce, many men develop a depressive episode.”
		Biological factors	7	10	“In my opinion, they are either actually genetically determined, i.e. hereditary gene structures.”
Admission into the mental health care system	Late initiation of treatment	Crisis situation	30	57	“And then because of all the overload at work and so on and then, yes, at some point, collapses.”
		Burden of dependents	6	10	“The burden of the women concerned is already very high. When they send the men to us.”
		Suicidality	10	19	“[…] they wait as long as they become suicidal, afterwards they will arrive at the hospital and even that often not voluntary.”
	precondition	Insufficient knowledge	10	20	“So I think that very few people say that they have depression, because they have no idea what [that] actually means. So they don’t know what it is.”
		Poor self-perception	14	17	“And the man hasn’t even noticed what’s been going wrong all these months and years.”
		Do not deal with mental issues	17	29	“And so men go somewhere with their buddies and of course emotions are rarely a topic of conversation.”
	Self-attribution of men	Accept help is related with weakness	22	41	“And to be able to manage things by yourself, otherwise you’re not a really a grown man.”
		Maintain self-worth	11	17	“And this [sense], if they are not needed, because they can’t do it any more, this means they quickly develop the feeling that they are no longer needed and worthless.”
		Sense of responsibility	15	20	“[...] In terms of the family situation – have situations begun to develop where they also have obligations, [where] they have to do what is expected, you know?”
	Stigma/ predjudices		10	21	“What would the village say if I was in a madhouse?”
	Structural barriers	Concern about future career	22	37	“Is he going to lose his position or his leadership position in the company? Because he has this condition, because it’s a condition that doesn’t persist for 2 weeks, but often lasts 10 or 12 weeks, and then they go to rehab and of course they become afraid of loss.”
		Knowledge of GPs	4	5	“Well, perhaps not all GPs are up to date on depression. And perhaps we have to think about what are, I would say specific, if there are any specific male symptoms that can be indicative of depression.”
	Intrinsic motivation	To function	6	6	“So, to recover just to be able to function again – that’s the main desire.”
		Partner/family	7	7	“[…] that you’d say, I want to do that for the sake of my family, or I want to continue to support my children and just want to be healthy for them.”
		Pension request	6	8	“[…] perhaps even a type of man who accepts this diagnosis very well or even desires it; these are people who are for example in their 50s or early 50s and have difficulties getting back into the job market and want a pension plan.”
	External motivation	General practitioner	7	9	“So, patients are often sent by their GP. Both men and women. GPs are often the first point of contact, […] and many patients first go to their GP as someone they can trust.”
		Colleagues & friends	2	2	“Then perhaps friends do it, or perhaps colleagues do it, if they react well.”
		Family	14	18	“So I guess if there’s a family that works reasonably well, then it’s likely that the partner or perhaps the parents or parents-in-law will say at some point that enough is enough. Now you have to do something about it.”
MHPsʼ views of male patients’ expectations	Expectations	Short treatment duration	12	18	“‘Short and quick’ has been said to me.”
		Complete recovery	7	7	“So men do expect this kind of complete recovery.”
		Medication	7	10	“And then it’s just clear, okay, it has to be medication.”
		Psychotherapy	2	4	“So because with the psychotherapeutic conversations many men somehow… then they would have to actually say something about themselves and so on.”
	Approaches	Sceptical about treatment	6	7	“And then they ask what the point is and say that they think they’re not making any progress.”
		Mechanical thinking	8	16	“You flip a switch and then you’re fine again.”
		Solution-oriented	8	10	“They say ‘What should I do? I’ll do it’.”
MHPsʼ mental health treatment aims	Psycho-education		23	57	“But an explanatory model is therefore very important. So, clarification and an explanatory model: why do I have this and what is it exactly?”
	Social environment		3	3	“So then I always try to make contacts.”
	Normalisation		18	27	“So, anything that brings a bit more stability somehow, a bit more insight into the situation as a whole, yes, that it is simply… that it is normal and that it can happen.”
	Distance oneself & Self-perception		4	9	“What I think is important is that I need to learn to distance myself if it concerns my health. In which situations am I overwhelmed? Or in which situations do I always continue to function but should start to say, Stop, stop, this far and no further?”

### MHPs’ descriptions of male patients

When asked to describe male patients with depression, most MHPs referred to a certain type of man (“typical” men) (Table 2). In contrast to this, some MHPs described “non-typical” men, usually after being prompted by the researcher. In contrast to the description of “typical” men, “non-typical” men were usually described with reference to female stereotypes (Table 2). However, not all participants reported on different types of men. A few MHPs explicitly denied the impact of gender as an important factor in the treatment of depression.

#### The “typical” men

MHPs described “typical” male patients as focused on gainful employment, committed to caring for their families and generally reluctant to discuss mental health issues. Regarding men’s focus on gainful employment, MHPs reported the existence of a strong work ethic and emphasized the importance of being (successfully) employed for most men. As noted, being responsible for the family was of key importance:

*“You have to feed your family. You have to do your job” (Social worker)*

From an MHP perspective, men are seen as being afraid of losing the ability to provide for their families. Faced with these demanding expectations, many men hesitate to show weakness:

*“Perhaps it’s harder for men to really show their weaknesses. That is, that they are just not the strong provider, who is always stable and always takes care of everything, but to be able to admit that sometimes something isn’t going well. I think it’s harder for men to do this.” (Psychologist).*

Consequently, this means that *“You also try to hide it for as long as possible, or you even try not to believe that you really have depression.” (Social worker).*

MHPs described men as typically not being familiar with conversations about emotions and mental health issues. Generally, men were assessed as having a poor understanding of mental health problems and mental health services and holding prejudices regarding them. In contrast, women were generally described as being more familiar with mental health issues:

*“But in terms of psychology, I think that’s a bit more the women’s domain, and they tend to accompany their children to the school counsellor or are the ones who go to educational counselling sessions and know that these things exist and talk about them with their female friends.” (Psychologist).*

In particular, older men, those living in rural areas, or those with a migration background were described as holding prejudices about mental health problems and treatment. In their description of “typical” men, most MHPs focused on male behaviours and attitudes. However, one MHP referred to the physical appearances of “typical” men by using masculine stereotypes including “*muscular*”, “*bald head*” and “*moustache*” (Table 2).

#### The “non-typical” men

Particularly when asked about differences between male patients, MHPs described a category of men which contrast with “typical” men in terms of lifestyle and mental health issues. MHPs characterized “non-typical” men as having positive attitudes towards mental health and treatment; for example, being aware of mental health issues and open minded about mental health treatment:

*“[ … ] men who have a better relationship to their feelings, who then also more easily show their feelings and talk about them.” (Social worker)*.

In contrast, some MHPs used more negative attributes such as “*wimp*” or “*softy*” to describe “non-typical” men (Table 2). At the same time, some MHPs distanced themselves from negative evaluations by using the phrase “*as the saying goes*” or quotation marks in their descriptions of such men:

*“And then there are the so-called ‘wimps’.” (Physician)*.

In their descriptions of “non-typical” men, some MHPs referred to female stereotypes such as being shy, talkative or passive (Table 2). When asked for differences between men, one participant stated, referring to “non-typical” men:

*“Well, they actually take on a ‘female role’.” (Physician).*

#### No gender variation

A few MHPs (5/33) consistently described male patients as not being significantly different from their female counterparts. In their view, it is individual characteristics that should play a role in mental health treatment:

*“So, something that is very upsetting for one person can also be stressful for another, but does not cause depression, so I think the causes are very diverse and lie partly in a susceptibility which is shaped by childhood experiences, personality traits and other factors and external pressures. So, [whether it’s] something very special or general, I can’t pin down right now. In principle, it really depends on the person.” (Psychologist).*

### MHPs’ theory of illness

MHPs mostly referred to psychosocial models in their explanations of the causes of depression and provided sociological explanations for the development of masculine ideals among men. According to MHPs, most men follow the ideal that demands they be successful in their work. They described men who relate pursuing a career to social approval:

*“[…] an unemployed man or a man who has problems at work is socially less valuable than a woman who doesn’t work.” (Physician).*

Accordingly, stressful working conditions and conflicts at work were reported as major causes for depression in men. Some MHPs (9/33) reported that men with narcissistic personalities appear to be particularly vulnerable to these pressures. However, MHPs emphasized that most men exhibit poor coping strategies or none at all. Extensive alcohol consumption or drug usage was reported as a form of self-medication.

MHPs discussed the development of masculine ideals with particular reference to socialisation and the internalization of gender norms. Strength, bravery and perseverance are described as typical gender norms for boys, while girls are raised to be needy:

*“So, we learn from an early age that men must somehow pull themselves together and are not allowed to cry. Dads are always saying “Indians don’t know pain”. When a woman or a little girl falls down, however, you run after her and say… well, you practically rescue her. I think it also has a bit to do with parenting.” (Physician).*

According to MHPs, most men are not used to communicating their needs. Instead, men seek to solve problems by themselves. MHPs described a consequence of this as being that men are not used to disclosing mental health problems to another person:

*“Until men are able to talk intimately, and here I include myself, there will still need to be a special context and a particular atmosphere, or otherwise somebody asks and really wants to know how one is, or the other person says something and then I also come out of my shell.” (Nurse).*

In comparison to men, women are commonly described as being “willing to provide personal information more easily and be more open” *(psychologist),* as well as talking with friends about their state of health, emotions and other intimate subjects:

*“From her state of mind to what tampon she [uses] and I don’t know what else.” (Nurse).*

As a consequence, female patients are more prepared to be open when in need of help.

Some MHPs discussed the real-world impact of changing expectations regarding gender roles in society. As one MHP put it, social change will result in changing attitudes towards men’s depression:

*“Yeah, in the old days, say 1988 or ‘91, when you talked about depression in men, that was something very negative for a man. That would have been like, ‘yes, he’s a real pussy, it’s no surprise. He just whines all day long and doesn’t manage anything and yes, he was always so bad at soccer back in the day, he didn’t get this and that done. And that’s the end result’. And nowadays it is more the case that it is a condition that is accepted, that it actually exists and that you aren’t able to help it.” (Nurse)*.

Another MHP posited that prevalence rates of depression among men and women will equalize in future because *“they [men, M.S.] can get depressed, too.” (Physician)*.

### Admission into the mental health care system

When asked about conditions surrounding the beginning of treatment in men, most MHPs reported that inpatient treatment is initiated at a late stage of the patient’s depression, usually after a crisis situation, suicidal ideation or attempted suicide:

*“Yeah, if they’re really down. Mostly when they’re really at their lowest point. Yes. And as I said before, there is nothing more serious than suicidal tendencies.” (Physician).*

Generally, most men were assessed as having scarcely any conception of mental health issues in general and poor self-perception regarding their own mental health. MHPs predominantly described men’s claimed ability to be able to solve problems by themselves. In their view, making use of depression treatment is associated with weakness and a loss of self-worth. As a consequence, most men hesitate to admit shortcomings linked to mental illness and exhibit delayed insight and readiness to start treatment. Around half of MHPs referred to men’s role as the family provider and their sense of responsibility towards their employer, both of which would be questioned by admitting the existence of depression and its treatment.

Some MHPs (10/33) reported a perceived stigma around depression as being a substantial reason for men to avoid treatment. In their view, it is particularly difficult for men to disclose the existence of depression in their professional and private environments compared to women. MHPs predominantly reported that most men are very concerned about the future of their careers. In addition, a minority of MHPs (4/33) explained a delay in treatment as being due to insufficient knowledge about “male depression” among general practitioners (GPs).

MHPs discussed the manifold role of the family in MH treatment: while conflicts with the partner or family as well as men’s desire to be a helpful family member are factors in men’s decision to seek treatment, partners and other family members were reported as being the most important persons to initiate treatment. Some MHPs (7/33) described men being referred for treatment by GPs and emphasized their significant role as patients’ first source of treatment information.

In the view of some MHPs (6/33), older men in particular hope that treatment for depression will result in a successful pension application. This might be explained with regards to feelings of being overworked or financial pressure in case of unemployment. Starting depression treatment is dependent on both intrinsic and external motivation in men. Some MHPs (5/33) referred to the desire in men to be able to “function” again. MHPs used this term very frequently throughout the interviews to describe men’s guiding principle in life. In their view, however, this attitude in men must be viewed somewhat critically:

*“Being functional every day just doesn’t work.” (Therapist).*

### MHPs’ views of male patients’ expectations

According to MHPs, most men tend to have high expectations with regard to MH treatment. As reported, male patients expect short treatment duration, high success rates in recovery and therapeutic sessions which are regarded as ‘reasonable’. Several MHPs (7/33) described men as being less interested in the process of recovery and more in the final outcome of treatment. Most men were reported as favouring a structured approach and practical advice in order to achieve treatment targets. Several MHPs (8/33) described the idea of mental health treatment in most men as being mechanical, using the analogy of repairing a car:

*“And what men like very much, but what you can’t always offer from a therapeutic point of view, is to know what the solution is. (laughs) You know? And that a solution can develop in the process, so to speak, can develop in the course of treatment, that is sometimes difficult to teach them. This mechanical way of thinking, that ok, we have a replacement part ready, the faulty part can now be removed and then the new part takes its place and I can function again as before. That is a kind of thinking that is somewhat obstructive to recovery.” (Psychologist).*

When asked about the conditions of the beginning of treatment, some MHPs described high rates of self-discharge after crisis intervention:

*“That means they always try to be discharged the day afterwards. When I think of the likelihood now, it’s around 50%, this likelihood.” (Physician).*

Pharmacotherapy is reported as being an important treatment option for most men. In particular, fewer worries regarding weight gain (when compared to women) and the reluctance to talk in psychotherapy are described as the main reasons for preferring drug-based treatment. Further, some MHPs (6/33) mentioned that medication is already understood as a form of treatment for physical complaints:

*“Medication is like... they are already familiar with that, they take it for their heart, or something similar. And yes, that means it fits with what they already know.” (Psychologist).*

Nevertheless, a few MHPs mentioned sexual dysfunction as a potential side effect of pharmacotherapy that makes men worried. MHPs described most men as being rather sceptical about therapeutic sessions that are not part of drug treatment but function as complementary treatment options:

*“So, as with occupational therapy, this idea of what, I have to do handicrafts now?! And like, what is the point of going for a walk, and do we really have to meet and talk every week?” (Physician).*

Some male patients are reported as showing a lack of self-initiative:

*“It’s the evil world’s fault. I don’t have to do anything about that.” (Nurse).*

One MHP reported feeling pressured because of patients’ high expectation in terms of treatment duration:

*“So I feel pressure as a therapist in these situations. (laughs) Yes.” (Psychologist).*

### MHPs’ mental health treatment aims

MHPs discussed the consequences for treatment against the background of normative expectations regarding the male gender. According to MHPs, psychoeducation and men accepting depression as a widespread mental illness are key goals for mental health treatment, with making patients understand their illness of primary importance. Compared to female patients, men *“definitely need more input from the psychotherapeutic side.” (Psychologist).*

About half of the participating MHPs (16/33) stated the aim of increasing the awareness in men that depression is no result of individual failure or frailty:

*“That it [is] not a weakness, but that it can happen. And that it exists and is relatively common.” (Psychologist)*.

According to MHPs, reflecting on working conditions, increasing self-perception and mindfulness are also required:

*“And then, of course, with the re-entry in the job market – what would also be important for men would be to say, okay, now you have just had to have this experience, how can I protect myself now? That’s not just about the work, but also what do I have to do in my spare time so that I don’t have to deal with being overworked anymore. How can I protect myself? What are these… what signals are there that I have to watch out for?” (Social worker).*

## Discussion

This study explored gender-specific treatment of depression in Germany from a professional perspective by exploring MHPs’ views on depression in men, the needs of male patients and approaches and treatment strategies regarding men with depression.

Most MHPs reported that male clients generally differ from female patients regarding knowledge of mental health issues as well as attitudes and expectations in terms of treatment. Men are considered as having a poorer understanding of mental health issues, higher expectations regarding the duration and outcomes of treatment, and mechanical conceptions of mental health treatment processes. At the same time, MHPs were found to describe basically two types of men: according to MHPs, the “typical” men is considered as in Iine with the ideal of hegemonic masculinity [36], including being strongly work-oriented, having breadwinner mentality and a reluctance to talk about mental health issues. Men considered as “typical” by MHPs were perceived as having work-related problems as a central cause for their depression. As a consequence, regaining professional status through employment was perceived to be significant for the recovery process.

In contrast, male patients who deviate from these patterns were considered as “non-typical” men and described by MHPs with reference to female stereotypes. Mental health problems among “non-typical” men were considered by MHPs as resulting from relationship problems or divorce. “Non-typical” men were perceived by MHPs as being more ready and open to talk about mental health issues, more willing to accept depression and to collaborate in treatment.

Based on their consideration of the differences between men and women with regard to knowledge, attitudes, and treatment expectations, some MHPs perceived a need to adapt their treatment strategies to the specific needs of male patients, including information regarding mental health processes and psychoeducation. In their regard, traditional male gender role orientations are associated with a refusal to seek help, a limited willingness to disclose symptoms, and the discontinuation of treatment. Accordingly, a majority of MHPs would prefer treatment strategies helping men with depression better understanding their mental health stress and encouraging them to rethink their expectations of male gender roles. MHPs’ suggestions of treatment strategies are in line with recommendations in the relevant literature relating to gender-specific treatment. It is recommended that MHPs address gender role expectations in men because these contribute to behaviour in men which is detrimental to their health [37, 38] and lead to men being reluctant to access depression treatment [39].

Some MHPs reported preferring mechanical explanatory models of depression or biological approaches regarding mental health in accordance with male patients’ conception of illness. In their illness conceptions of depression, most MHPs relied on psychosocial explanations rather than biological factors. Focusing on the specific needs of older men with depression, Apesoa-Varano and colleagues [30] found that MHPs pursue three approaches in treating depression. MHPs who pursue (1) an indirect approach focus on (particularly somatic) symptoms and substitute the term “depression” with similar terms such as “stress”. This approach is explained with regard to the reservations expressed by this group of patients towards being labelled “depressed” due to male gender norms and the stigma surrounding depression [30]. Also, they described (2) a step-by-step introduction of the term “depression” after an initial confidence-building phase which is identical with the indirect approach. However, some professionals reported pursuing a (3) direct approach in order to give patients a reality check, using the term “depression” directly and unambiguously [30].

Our results revealed contradicting gender stereotypes and sometimes even stigmatising attitudes among MHPs. On the one hand, they considered “typical” men with a traditional masculinity orientation as reluctant against treatment, on the other hand, they regarded “non-typical” men as being open-minded towards mental-health issues but at the same time characterized them in a derogatory manner with female stereotypes such as being a “wimp” or “soft”. Men with depression and behavioural patterns diverging from the ideal of hegemonic masculinity might experience discriminating and stigmatising attitudes towards their masculinity as well as their mental illness [40, 41]. These results correspond to those of Oute and colleagues [31] who found that patients who were characterized as being weak, dependent and sensitive were described by MHPs as persons who are more able to talk about mental health issues, having more profound insight into their own mental health problems and being more willing to accept professional help [31]. In contrast, patients who were characterised as active, autonomous and self-reliant were described as persons who hardly can accept mental health problems and showing consecutive symptoms such as alcohol consumption and substance abuse [31]. Patients with atypical behavioural patterns were assessed by MHPs as being deviant patients who are more difficult to treat. Consequently, these patients are at greater risk of being excluded from psychiatric treatment and referred to other institutions, instead of receiving adequate treatment for their depression [31]. Recommendations in relevant literature to avoid treatment inequity based on gender stereotypes include awareness of preconceptions towards men and women with depression [31, 42, 43] to ensure that MHPS’ own prejudices don’t lead to bias in the recognition and consideration of patients’ needs [31]. Some literature shows that behaving in accordance with traditional masculinity may also be a valuable resource for men to get through depression [43, 44] and suggest that building on the strengths of each form of masculinity might raise attractivity to male patients [29]. Therefore, the discussion of traditional masculinity as a risk factor regarding mental health issues in men represents a one-dimensional understanding of masculinity and underestimates the positive aspects of traditional masculinity in mental health treatment. Therefore, a more differentiated understanding on the diverse implications of masculinity for mental health would be essential with regards to future interventions, for which further research that incorporates a critical examination of traditional masculinity is required [45]. Interventions to reduce mental illness stigma in health professionals may need to run a multilevel approach, adjusted to groups of health professionals with varying characteristics of stigmatising attitudes [46, 47] personality characteristics and job strain of MHPs [48].

### Strengths and limitations

To our knowledge, this is the first qualitative study on MHPs’ perspectives regarding the impact of male gender in depression treatment in Germany. Several strengths and limitations of the study should be discussed: Firstly, the sample of MHPs was limited to a number of psychiatric hospitals in a rural area. This might lead to rather traditional concepts of gender in comparison to urban areas. Therefore, as well as in all qualitative studies, results are not statistically representative and cannot be generalized to all MHPs. At the same time, we are confident to describe the general perspective of MHPs due to the variety of psychiatric hospitals which reflect the mental health care supply system in Germany. To explore a broad range of expertise and multifaceted findings, we further included MHPs with different professional backgrounds. Secondly, we need to consider a selection bias of participants due to the assumption that participants showing a special concern in this topic very likely agree to participate in a study. However, we could not find a systematic tendency of participants’ answers in our analysis.

Thirdly, it should be considered that both interviewers were female. Against the background of interactionism, we should take into account that male and female participants might answer in a different way depending on the gender of the interviewer. Thus, we must take into account the possibility that the interviewers’ gender had an impact on participants’ answers and the way in which certain issues were addressed.

## Conclusion

To improve mental health among men and to contribute to the development of gender-specific mental health services, awareness of the role of gender and its implications for mental health treatment should be an integral part of MHPsʼ education and the everyday practice of MH treatment.

## Supplementary information

**Additional file 1: Figure 1.** Interview guide

## Data Availability

Data will not be shared due to difficulties in the anonymization of qualitative data. Access to data will be granted to researchers for appropriate use.

## Abbreviations

MHPs: Mental Health Professionals
MH: Mental Health
