# Supplementary material for: Mental health professionals view about the impact of male gender for the treatment of men with depression - a qualitative study
Source: BMC Psychiatry. 2020 Jun 3;20:276. doi: 10.1186/s12888-020-02686-x (PMC7268222; doi:10.1186/s12888-020-02686-x)
Supplement: Supplementary file 1 — Additional file 1: Figure 1. Interview guide [file 12888_2020_2686_MOESM1_ESM.docx]

| Theme | | Impulse | Further Enquiry | Background |
| --- | --- | --- | --- | --- |
| Conceptions of illness | | In the light of your experience: What are the reasons that men come down with depression? | What are the main differences between men and women suffering from depression?  (consider for all questions) | - risk- and protective   factors   - biological factors - social factors |
| Treatment start | Motivation | When do men suffering from depression start inpatient treatment? |  | - reasons - determinants - role of the family - implication of the   illness on different areas of  life   - self-stigma - stigma experiences in living   environment   - differences in symptoms   between men and women   - gender-specific   questionnaires |
|  | Symptoms |  | How do male patients with depression behave when they start inpatient treatment? |  |
|  | Time |  | At what time during the progression of illness do men start depression treatment? |  |
| Treatment | Performance | How do male patients with depression perform during treatment/care? |  | - behaviour |
|  | Needs | What do you think is required during treatment of male patients with depression? |  |  |
|  | Dealing with  the diagnosis |  | How do male patients with depression deal with the diagnosis? | - generations - milieu/class - ethnics - migration background - acceptance of diagnosis   (during treatment process) |
|  | Language |  | What is your experience about how mental health professionals should talk with men about depression? | - depression named (at what   time during the process of treatment )   - reasons for different   approaches/procedures |
|  | Social network | What is your opinion about the importance of the social network during treatment of depression in men? | Importance of the family for male patients with depression?  Importance of friends for male patients with depression?  Importance of attitudes of the employer/ work environment? | - including family in the   treatment process   - paternity - violence - reputation - fear of failure - fear not to meet requirements |
|  | Treatment aims |  | What do male patients with depression expect from/during treatment?  What are your key goals in mental health treatment? | - return to work - treatment method:   medication/psychotherapy   - duration of treatment |
| Services | | Are there gender-specific services for men with depression at the hospital? | Yes: What kind of?  No: What kind of services would be reasonable? | - single-sex groups/ mixed-sex groups |
| Gender of MHP | | Does the gender of the Mental Health Professional have an impact in depression treatment in men? | Yes: What kind of? | - stigma - shame - expectations |
| Additional  issues | | Is there anything else with regard to treatment of depression in men you want to talk about? |  |  |
